# Malnutrition-Sarcopenia Syndrome in older adults: Causes, consequences, and countermeasures

**DOI:** 10.17179/excli2026-9426

**Published:** 2026-06-10

**Authors:** Carina O. Walowski, Kristina Norman

**Affiliations:** 1 Department of Nutrition and Gerontology, German Institute of Human Nutrition Potsdam-Rehbrücke, 14458 Nuthetal, Germany; 2 Department of Geriatrics and Medical Gerontology, Charité-Universitätsmedizin Berlin, Corporate Member of Freie Universität Berlin and Humboldt-Universität zu Berlin, 13347 Berlin, Germany; 3 Institute of Nutritional Science, University of Potsdam, 14458 Nuthetal, Germany; 4 German Center for Cardiovascular Research (DZHK), Partner Site Berlin, 10115 Berlin, Germany

**Keywords:** sarcopenia, malnutrition, protein supplementation, resistance training, older age

## Abstract

Whether the Malnutrition-Sarcopenia Syndrome (MSS) represents a distinct clinical entity or simply describes severe malnutrition with prominent muscle wasting remains debated. Nevertheless, the coexistence of inadequate nutritional status and severe loss of skeletal muscle mass, strength, and function creates clinically significant challenges that warrant focused attention. The causes of MSS involve a vicious cycle driven by insufficient dietary intake, inflammation, hormone deficiency as well as physical inactivity. In a bidirectional relationship, malnutrition accelerates muscle protein breakdown and impairs synthesis, while sarcopenia reduces functional capacity and potentially leads to decreased physical activity and loss of independence. The consequences are substantial and include accelerated functional decline and delayed convalescence, reflected by prolonged hospital length of stay and higher rates of increased mortality compared to single conditions. Effective countermeasures require integrated interventions addressing both components simultaneously: adequate protein and energy intake, and vitamin D, combined with progressive resistance training, treatment of underlying diseases, and medication optimization. This multidisciplinary approach demonstrates synergistic benefits that exceed addressing either condition alone. Regardless of whether MSS constitutes a unique syndrome, recognizing this clinical pattern serves the pragmatic purpose of identifying vulnerable older adults who require comprehensive, simultaneous nutritional and functional interventions to break the vicious cycle and improve outcomes.

See also the graphical abstract(Fig. 1).

## 1. Introduction

Aging is accompanied by the accumulation of conditions contributing to progressive health deterioration and loss of independence. Among these, malnutrition (impaired nutritional status) and sarcopenia (age-associated loss of muscle mass, strength, and function), represent two highly prevalent and clinically significant disorders, often co-occurring in older adults (Vandewoude et al., 2012[118]). Pathophysiologically, both conditions are closely interconnected, sharing multiple underlying mechanisms. These are driven by age-related intrinsic changes such as altered energy metabolism, decline in anabolic hormones, insulin resistance (IR), low-grade inflammation, and “anorexia of aging” and are further compounded by extrinsic and behavioral factors including physical inactivity, chronic disease burden, and polypharmacy (Vandewoude et al., 2012[118]; Norman et al., 2021[89]; Wiedmer et al., 2021[128]).

Beyond these shared mechanisms, malnutrition and sarcopenia can mutually reinforce each other. Insufficient energy and protein intake can accelerate the loss of muscle mass and function (Landi et al., 2019[70]), whereas sarcopenia may further compromise nutritional status, creating a vicious cycle, in which each condition exacerbates the progression of the other. Reflecting this close interplay, the Global Leadership Initiative on Malnutrition (GLIM) has incorporated low muscle mass as one of the phenotypic diagnostic criteria for malnutrition (Cederholm et al., 2019[18]).

To capture the clinical overlap between these conditions, Vandewoude and colleagues (2012[118]) introduced the term of “Malnutrition-Sarcopenia Syndrome” (MSS), defined as the concurrent presence of malnutrition together with accelerated age-associated decline in skeletal muscle mass and strength, and/or physical performance. Whether MSS constitutes a distinct clinical syndrome or rather represents the frequent co-occurrence of two closely related entities remains a subject of ongoing debate. Nevertheless, skeletal muscle is the most affected organ in MSS, which is particularly critical in older adults, as muscle loss is rarely recovered spontaneously.

Systematic prevalence estimates for MSS are limited, but epidemiological evidence across various populations and settings has demonstrated substantial coexistence. In community-dwelling older adults at nutritional risk, approximately 35 % were found to have concurrent sarcopenia (Taani et al., 2021[114]). Hospitalized older adults showed even higher rates, with a systematic review and meta-analysis reporting that 41.6 % of patients presented with both conditions, and that malnutrition was associated with an approximately fourfold increased likelihood of sarcopenia (Odds Ratio (OR): 4.06, 95 % Confidence Interval (CI): 2.43-6.80, p < 0.001) (Ligthart-Melis et al., 2020[73]). In geriatric rehabilitation inpatients, about 13 % manifested concurrent malnutrition and sarcopenia, with malnutrition doubling the risk of severe sarcopenia (OR: 2.07, 95 % CI 1.13-3.81, p = 0.019) (Verstraeten et al., 2021[120]). Evidence from disease-specific cohorts further highlights the prevalence of MSS in high-risk populations, affecting 18.2 % of patients undergoing hemodialysis (Wang et al., 2025[126]) and 17.5 % of patients with gastric cancer following radical gastrectomy (Chen et al., 2022[23]).

Both malnutrition and sarcopenia are independently associated with adverse health outcomes, including increased morbidity and mortality, reduced quality of life, and higher rates of rehospitalization, prolonged hospital stays, and increased healthcare costs (Vandewoude et al., 2012[118]). Emerging evidence indicates that their coexistence may exert additive or even synergistic detrimental effects on health, exceeding the impact of either condition alone and resulting in accelerated functional decline, higher complication rates (Chen et al., 2022[23]), and increased mortality (Hu et al., 2017[58]; Chen et al., 2022[22]; Gümüşsoy et al., 2021[52]). Consequently, the clustering of malnutrition and sarcopenia poses a major healthcare challenge, underscoring the critical need of early identification and management. Yet, to date, no specific diagnostic criteria or evidence-based therapeutic strategies exist for MSS revealing a critical gap in the care of older adults.

The aim of this review is not to establish valid diagnostic or therapeutic guidelines for MSS, but rather to (i) highlight the changes in aging skeletal muscle predisposing to MSS, (ii) synthesize current knowledge on shared and interacting mechanisms of malnutrition and sarcopenia, (iii) examine their synergistic clinical consequences, and (iv) discuss potential strategies for early identification and management, while drawing attention to critical gaps in clinical care and research.

## 2. Etiology

### 2.1 Age-related intrinsic factors: Impact on skeletal muscle

Aging is associated with profound remodeling of skeletal muscle composition, which is primarily driven by the loss of type II (fast-twitch) fibers (Cade and Yarasheski, 2006[14]; Domingues-Faria et al., 2014[40]; Phu et al., 2015[93]; Cho et al., 2016[25]). Declines in mRNA expression of myosin heavy chain isoforms IIa and IIx may contribute to this preferential loss of fiber type, and, in turn, to the reductions in muscle strength observed in sarcopenia (Cade and Yarasheski, 2006[14]).

In addition, advancing age is related to a reduced number of motor units (Cade and Yarasheski, 2006[14]), reflecting decreases in motor neuron number and axonal cell body size (Kawamura et al., 1977[64]), which may impair neuronal skeletal muscle activation (Kido et al., 2004[65]) and thereby further promote age-related muscle dystrophy (Wiedmer et al., 2021[128]).

Satellite cells, the resident stem cells of skeletal muscle critical for growth and regeneration, decline in number per myofiber, along with its regenerative capacity, as age advances (Cade and Yarasheski, 2006[14]; Domingues-Faria et al., 2014[40]), particularly those associated with type II fibers (Domingues-Faria et al., 2014[40]).

These changes are accompanied by systemic endocrine dysregulation, characterized by altered skeletal muscle protein turnover, with reduced muscle protein synthesis (MPS) rather than elevated muscle protein degradation (MPD) being the primary driver (Cade and Yarasheski, 2006[14]). With advancing age, MPS declines by approximately 30 %, mainly due to lower synthesis rates of mixed muscle proteins (myofibrillar, mitochondrial, and sarcoplasmic fractions) (Cade and Yarasheski, 2006[14]).

The growth hormone (GH)/insulin-like growth factor-1 (IGF-1) axis constitutes a main anabolic regulator of skeletal muscle. IGF-1 stimulates MPS and myogenic differentiation primarily via the activation of the phosphoinositide 3-kinase/protein kinase B/mammalian target of rapamycin complex 1 (PI3K/Akt/mTORC1) cascade (Velloso, 2008[119]; Yoshida and Delafontaine, 2020[135]), whereas age-related declines in GH (~14 % per decade post-puberty; somatopause) and circulating IGF-1 levels attenuate this signaling, favoring net catabolism (Sattler, 2013[101]) (Figure 2(Fig. 2)). Older adults with sarcopenia have been observed to exhibit lower circulating levels of GH, IGF-1, and the IGF-1 splice variant mechano-growth factor (MGF) compared to age-matched controls (Bian et al., 2020[10]). Multivariate analyses confirmed IGF-1 and/or MGF as independent predictors of reduced appendicular skeletal (Bian et al., 2020[10]) and whole-body muscle mass (Walowski et al., 2023[125]), with lower IGF-1 levels also correlated to higher intramuscular fat infiltration in the lumbar region of multifidus and erector spinae muscles (Walowski et al., 2023[125]), reduced handgrip strength (HGS) (Van Nieuwpoort et al., 2018[117]; Walowski et al., 2023[125]), and impaired physical performance (Van Nieuwpoort et al., 2018[117]).

Closely linked to the GH/IGF-1 axis, insulin represents a further key anabolic regulator in skeletal muscle, activating PI3K/Akt/mTORC1 signaling to stimulate protein synthesis while inhibiting forkhead box O-mediated proteolysis (Sakaguchi, 2024[100]) (Figure 2(Fig. 2)). With aging, myocytic insulin sensitivity declines markedly, impairing both glucose and amino acid uptake. Skeletal muscle accounts for ~80 % of insulin-stimulated whole-body glucose disposal (DeFronzo and Tripathy, 2009[34]; Merz and Thurmond, 2020[79]), yet older adults exhibit a 30-35 % reduction compared with younger individuals (Fink et al., 1986[47]), highlighting skeletal muscle as the primary locus of age-related IR. This anabolic resistance may stem from multiple factors blocking PI3K/Akt/mTORC1 signaling, including chronic low-grade inflammation, ectopic lipid accumulation (ceramides/diacylglycerols), mitochondrial dysfunction, endothelial impairment, and reduced autophagy (Shou et al., 2020[108]).

Similarly, age-related declines in circulating sex steroids, including testosterone in men (ap-proximately 1-1.6 % annually for total and 2-3 % for free testosterone from midlife) and es-trogens in women during menopause (Feldman et al., 2002[45]; Burger et al., 2007[13]), are associated with sarcopenia and may contribute to it via reduced anabolic signaling, impaired MPS, and diminished satellite cell activation and proliferation, while upregulating proteolytic signaling pathways. Concurrent fat accumulation may further promote tumor necrosis factor-α (TNF-α) and interleukin-6 (IL-6) signaling, oxidative stress, and mitochondrial dysfunction, collectively shifting skeletal muscle toward net protein catabolism (Huang and Wang, 2021[59]; Shigehara et al., 2022[106]). Notably, most mechanistic insights into these pathways derive from preclinical in vivo and in vitro models.

Cross-sectional analyses have shown that community-dwelling older men with sarcopenia exhibit lower total and free testosterone levels compared with peers without sarcopenia (Diago-Galmés et al., 2021[39]). Multivariate logistic regression further indicated an association between higher circulating free testosterone and a lower prevalence of sarcopenia in both men and women (Shin et al., 2021[107]). Large epidemiological studies have linked reduced sex steroid concentrations with declines in muscle mass, strength, and physical performance (Vitale et al., 2016[121]). However, longitudinal findings remain inconsistent, as several investigations failed to confirm a clear predictive relationship between baseline testosterone levels and subsequent muscle decline (Roy et al., 2002[99]; Cawthon et al., 2009[16]; Vitale et al., 2016[121]). Evidence regarding circulating estrogens in postmenopausal women is comparatively scarce, limiting firm conclusions about their role in sarcopenia development.

Parallel to the decline in anabolic hormones, aging is accompanied by “inflammaging”, a sterile, chronic low-grade inflammatory state characterized by elevated pro-inflammatory cytokines such as TNF-α and IL-6 as well as increased levels of C-reactive protein (CRP) (Franceschi et al., 2000[48]). These mediators have been implicated in the dysregulation of muscle protein turnover, however, the precise mechanistic links in human sarcopenia are not yet fully clarified (Pan et al., 2021[90]). Evidence indicates that pro-inflammatory cytokines promote muscle loss by activating proteolytic pathways such as the ubiquitin-proteasome system and by impairing anabolic signaling mediated by insulin and IGF-1 (Ji et al., 2022[61]) (Figure 2(Fig. 2)).

In population-based cohorts, older adults with sarcopenia exhibited higher circulating IL-6 and TNF-α concentrations compared with controls (Bian et al., 2017[11]; Kamper et al., 2021[62]; Zhao et al., 2021[137]). In a longitudinal study of 986 individuals followed over three years, elevated IL-6 (> 5 pg/mL) and CRP (> 6.1 µg/mL) independently conferred a two- to threefold increased risk of > 40 % decline in HGS (Schaap et al., 2006[102]). Similarly, among nonagenarians, higher IL-6 and CRP levels were associated with lower HGS and reduced Barthel Index scores, underscoring the detrimental impact of inflammatory burden on physical function in very old adults (Tiainen et al., 2010[115]). Chronic low-grade inflammation in older adults has further been linked to central appetite dysregulation, as elevated cytokines such as TNF‑α and IL‑1β can act on appetite-regulating regions of the central nervous system (Morley et al., 1999[84]; Brown and Bradford, 2021[12]). The resulting reduction in hunger and increased early satiety, collectively termed as “anorexia of aging”, leads to insufficient energy and protein intake and thereby increases the risk of both quantitative and qualitative malnutrition, ultimately facilitating the development of sarcopenia (Wysokiński et al., 2015[131]; Cox et al., 2019[29]) (Figure 2(Fig. 2)).

Within the hallmarks of aging framework (López-Otín et al., 2013[75]), disrupted proteostasis, mitochondrial dysfunction, and inflammatory dysregulation have also been proposed as key mechanisms underlying sarcopenia (Wiedmer et al., 2021[128]).

Most importantly, emerging evidence indicates that age-related epigenetic modifications, particularly DNA methylation drift at cytosine-phosphate-guanine (CpG) dinucleotides, may modulate gene-regulatory programs in human skeletal muscle and thereby contribute to sarcopenia pathogenesis (Figure 3(Fig. 3)) (Voisin et al., 2021[122]; Antoun et al., 2022[2]). In a large epigenome-wide association study meta-analysis (n = 908 skeletal-muscle methylomes from individuals aged 18-89 years), Voisin et al. identified hypomethylation at atrophy-promoting genes, accompanied by an increase in gene expression for histone deacetylase 4 and an increase in protein expression for atrogin-1, while most differentially methylated genes did not alter at the mRNA or protein level but were enriched among genes showing age-related differential transcript and protein expression (Voisin et al., 2021[122]). Complementing these findings, genome-wide DNA methylation profiling (850,000 CpGs) revealed a globally hypermethylated profile in aged human skeletal muscle compared with young adult tissue, with hypermethylated CpG sites enriched in pathways central to muscle mass and function, including PI3K/Akt/mTOR, p53, axon guidance, and Hippo signaling, and across homeobox gene clusters critical for myogenesis and musculoskeletal development (Turner et al., 2020[116]). Moreover, in vastus lateralis muscle biopsies from older men, Antoun et al. observed widespread differential methylation between individuals with and without sarcopenia and identified additional differentially methylated CpGs (dmCpGs) associated with its component measures (grip strength, appendicular lean soft tissue index, and gait speed). Sarcopenia-associated dmCpGs substantially overlapped with those linked to each component and were enriched in genes involved in myotube fusion, oxidative phosphorylation, and voltage-gated calcium channels, suggesting that specific epigenetic alterations may contribute to age-related declines in muscle function (Antoun et al., 2022[2]). Additionally, in peripheral blood, lower fibroblast growth factor 2 levels (FGF2_30) were associated with both the risk and severity of sarcopenia in older adults, with methylation levels below 0.15 conferring approximately ninefold higher odds of sarcopenia compared to those with higher methylation levels (Li et al., 2024[72]).

Furthermore, DNA methylation patterns are highly responsive to sarcopenia risk-associated factors including inflammation (Yamashita et al., 2019[132]) and physical inactivity (Nalli et al., 2026[86]) as well as malnutrition with deficits in energy, protein (methyl-donors), and micronutrients (Hussain et al., 2026[60]) (Figure 3(Fig. 3)).

Taken together, epigenetic alterations may represent a critical frontier in the pathophysiology of MSS, suggesting a mechanistic link between lifestyle- and disease-associated factors and the long-term dysregulation of gene expression that drives skeletal muscle atrophy and metabolic dysfunction in the context of aging.

### 2.2 Dietary factors

Current evidence indicates that protein requirements are generally increased in older adults, and that meeting these needs is essential for preventing both age-related malnutrition and sarcopenia (Bauer et al., 2013[3]; Deutz et al., 2014[38]). Recommendations for protein intake are primarily derived from the concept of anabolic resistance, which refers to an attenuated responsiveness of aging skeletal muscle to anabolic stimuli. Key mechanisms contributing to anabolic resistance include increased splanchnic sequestration of amino acids, reduced postprandial delivery of amino acids to the periphery, lower postprandial muscle perfusion, impaired muscle uptake of dietary amino acids, blunted anabolic signaling pathways for protein synthesis, and age-related declines in digestive and absorptive function (Deutz et al., 2014[38]). However, protein intake among community-dwelling older adults is often found to fall below current recommendations (Hengeveld et al., 2020[55]).

Inadequate energy intake promotes a negative energy balance, enhancing the utilization of amino acids for oxidative energy production rather than for MPS and thereby limiting the effectiveness of dietary protein for maintaining muscle mass. Supporting this, data from 940 men and 1,324 women aged ≥ 65 years in the Fourth Korea National Health and Nutrition Examination Survey showed significantly lower energy intake in participants with sarcopenia (Kim et al., 2014[66]).

According to the underlying malnutrition type, the dynamics of protein catabolism can differ, with disease-related malnutrition leading to accelerated MPD and rapid loss of skeletal muscle mass, whereas age-related malnutrition is associated with a slower but progressive decline in muscle mass (Lengelé et al., 2021[71]; Norman et al., 2021[89]).

Micronutrient deficiencies represent an additional and often underrecognized component of malnutrition in older adults, with emerging evidence linking low micronutrient status to impaired muscle health and functional decline (Robinson et al., 2021[97]; Bhattacharya et al., 2022[9]). In particular, suboptimal status of B-vitamins has been associated with impaired skeletal muscle function, including reduced gait speed and lower HGS, potentially reflecting disturbances in energy metabolism, mitochondrial function, and neuromuscular integrity. Vitamin D deficiency is related to muscle pain and weakness, underscoring its role in MPS, calcium homeostasis, and contractile function. Furthermore, inadequate intake or status of minerals such as magnesium, calcium, iron, selenium, and zinc has been associated with impaired excitation-contraction coupling, compromised mitochondrial energy production, reduced antioxidant defense capacity, and diminished neuromuscular performance (Robinson et al., 2021[97]; Bhattacharya et al., 2022[9]), thereby amplifying the negative impact of low protein and energy intake on muscle mass and function. Most of the current evidence, however, arrives from animal studies or observational cohorts. Therefore, large‑scale randomized controlled trials are necessary to confirm these findings and to lay the foundation for the potential adoption of multi‑nutrient supplementation strategies in the management of sarcopenia (Bhattacharya et al., 2022[9]).

Taken together, inadequate dietary intake of energy and protein can initiate malnutrition‑related sarcopenia, while progressive muscle loss in turn impairs physical function and independence, making activities such as food shopping, cooking, and meal preparation more difficult and thereby reinforcing a self‑perpetuating cycle of malnutrition and sarcopenia.

### 2.3 Physical inactivity and immobilization

Habitual physical activity declines progressively with age promoting muscle atrophy as well as metabolic and functional impairment (Milanović et al., 2013[81]; Wall et al., 2013[124]; McPhee et al., 2016[78]). Among older adults, meta-analyses demonstrated a 1.7 to 2-fold higher prevalence of sarcopenia in individuals with low and no regular activity compared to active counterparts (Steffl et al., 2017[110]; Gao et al., 2021[50]). Prospective data (n = 2,309) further revealed a 5-year sarcopenia incidence of 14.8 % among the least active versus 9.0 % in the most active (Mijnarends et al., 2016[80]). Continuous exercise from early adulthood through old age was correlated with higher muscle mass (standard deviation (SD) = 0.24, p < 0.001), HGS (SD = 0.18, p < 0.05), and reduced clinically relevant low muscle mass risk in men (OR = 0.36, p < 0.01) (Eibich et al., 2016[42]), underscoring the critical importance of lifelong physical activity maintenance for sarcopenia prevention.

The most commonly employed models to study muscle disuse in humans have been bed rest and limb immobilization/suspension. Prolonged disuse (> 10 days) has been shown to induce rapid skeletal muscle atrophy at approximately 0.5-0.6 % of total muscle mass per day, with strength declining 0.3-4.2 % daily. These changes were primarily attributed to reductions in post-absorptive and post-prandial MPS, while protein breakdown contributed minimally (Wall et al., 2013[124]). Reduced activity further lowers total energy expenditure, which can suppress appetite and dietary intake. Moreover, inactivity is associated with IR and a pro‑inflammatory state, both of which can further impair MPS, promote muscle atrophy, and disturb appetite, energy balance, and nutrient metabolism.

Of potentially greater long-term relevance for the onset of sarcopenia and malnutrition may be the cumulative burden of recurrent short disuse episodes, such as brief hospitalizations or home-based recovery periods due to injury or illness. Successive events, associated with transient increases in MPD and reductions in synthesis, are thought to accumulate over the lifespan, driving net muscle loss with aging (Wall et al., 2013[124]). This effect is exacerbated by delayed and often incomplete recovery in older adults (Suetta et al., 2009[113], 2013[112]).

### 2.4 Disease and polypharmacy

Acute stressors such as infections, surgery, or trauma trigger a pronounced hyperacute stress response. This response is characterized by elevated pro-inflammatory cytokines, increased release of corticosteroids and catecholamines, insulin and growth hormone resistance, bed rest, and reduced or absent food intake, which together rapidly deplete energy and protein stores and accelerate tissue breakdown (Cederholm et al., 2017[17], 2019[18]). Chronic diseases, including type 2 diabetes mellitus, chronic obstructive pulmonary disease, or chronic kidney disease, act through largely similar mechanisms, but are distinguished by persistent low‑grade inflammation, which, together with altered metabolic demands, sustain protein-energy imbalance and progressive muscle wasting over time. Non-inflammatory conditions, including neurological and psychiatric disorders, cognitive impairment, dementia, and gastrointestinal diseases, compromise nutritional status through appetite dysregulation, malabsorption, and functional dependence (Cederholm et al., 2017[17], 2019[18]).

Beyond these disease-related drivers of malnutrition and sarcopenia, polypharmacy has emerged as an additional key factor in older adults, given the high prevalence of multiple medications. Polypharmacy and sarcopenia are strongly associated in older adults, with those affected by sarcopenia exhibiting a 65 % higher prevalence of polypharmacy and using, on average, 1.39 more medications than their counterparts without sarcopenia (Prokopidis et al., 2023[95]). However, predominantly cross-sectional study designs preclude conclusions regarding causality, direction of causality, and underlying mechanistic links. Nevertheless, multiple biological mechanisms implicated in sarcopenia may also drive its association with polypharmacy, including medication-induced mitochondrial dysfunction, impaired muscle perfusion, electrolyte imbalances, hormonal dysregulation, and acid-base disturbances (König et al., 2018[68]). Likewise, several commonly prescribed drug classes exert direct adverse effects on body composition: proton pump inhibitors, for instance, have been shown to be negatively associated with circulating IGF-1 levels (Maggio et al., 2014[76]); statins, glucocorticoids, and certain antiepileptic, neuroleptic, and antidepressant drugs are known to cause muscle toxicity, while others (e.g., beta blockers, non-steroidal anti-inflammatory drugs) potentially induce detrimental metabolic effects (König et al., 2018[68]). Chemotherapy and cytotoxic anticancer drugs can further directly promote muscle atrophy by suppressing protein synthesis, inducing oxidative damage, depleting cellular energy, and triggering apoptotic or necrotic cell death (Schiessel and Baracos, 2018[103]). Medications may also blunt acute exercise-induced signaling by suppressing key transcription regulators such as mitogen-activated protein kinases and nuclear factor-kappa B (Markworth et al., 2014[77]; Prokopidis et al., 2023[95]), both essential for skeletal muscle adaptations (Kramer and Goodyear, 2007[69]), potentially attenuating strength gains and hypertrophic responses (Lilja et al., 2018[74]).

Furthermore, polypharmacy contributes to sedentary behavior and reduced physical activity, with the likelihood of inactivity increasing alongside the number of medications (Heseltine et al., 2015[56]; de Souza et al., 2023[32]). It is also linked to adverse outcomes such as falls, disability, and impaired physical function, which can further limit activity (de Souza et al., 2023[32]). In addition, older adults taking multiple medications are more vulnerable to drug-related side effects that compromise mobility, including reduced walking speed from anticholinergics, myalgia or muscle weakness induced by statins and corticosteroids, and sedation, dizziness, or orthostatic hypotension from benzodiazepines and antihypertensives (de Souza et al., 2023[32]).

Finally, various types of drugs can induce anorexia due to their side effects contributing to impaired nutritional status or malnutrition (Heuberger and Caudell, 2011[57]; Gálvez et al., 2012[49]; Kok et al., 2022[67]). Polypharmacy further compromises nutrient bioavailability, with deficiency risk increasing with the number and duration of treatment (Zanetti et al., 2023[136]). Undertreatment is also a common issue in older adults with polypharmacy, particularly regarding micronutrients such as vitamin D and calcium, whose insufficiency may exacerbate sarcopenia risk and progression (Chau et al., 2016[21]).

## 3. Consequences

The consequences of malnutrition and sarcopenia are well established impacting functional and clinical outcomes as well as survival (Vandewoude et al., 2012[118]; Cruz-Jentoft et al., 2019[31]; Norman et al., 2021[89]; Beaudart et al., 2025[4]). When malnutrition and sarcopenia coexist as MSS, their effects may interact synergistically rather than additively, creating a vicious circle where malnutrition accelerates loss of skeletal muscle mass and sarcopenia worsens nutritional status, resulting in more rapid decline, more complications, and a higher mortality rate. Sarcopenia-related loss of muscle mass, strength, and function compromises physical performance and mobility, impairing balance, gait stability, and the ability to perform basic activities of daily living such as rising from a chair, climbing stairs, or walking. This increases the risk of frailty as well as falls and subsequent fractures (Yeung et al., 2019[134]). Malnutrition may accelerate this process by decreased availability of protein and other nutrients as well as by promoting catabolic inflammatory responses (Norman et al., 2021[89]). While existing evidence on malnutrition (Eckert et al., 2021[41]; Moon et al., 2023[82]; Chiavarini et al., 2024[24]) and sarcopenia (Beaudart et al., 2017[7]; Yeung et al., 2019[134]; Mu et al., 2026[85]) independently suggests that MSS would substantially increase the risk of frailty, falls, and (clinical outcomes after) fractures in community-dwelling older adults, prospective observational studies systematically evaluating these specific functional outcomes in well-defined populations with concurrent malnutrition and sarcopenia are still lacking.

The synergistic effect is, however, evident in acute or chronic disease and in traumatic injury with the risk of prolonged hospital length of stay due to delayed convalescence or complications (Correia and Waitzberg, 2003[28]). Functional recovery is similarly compromised, as malnutrition profoundly impairs wound healing (Demarest-Litchford et al., 2024[35]), tissue repair and muscle homeostasis, and affects immune function (Kawakami et al., 1999[63]; Schneider et al., 2004[104]), while pre-existing sarcopenia limits physical capacity even before injury. Postoperatively, these deficits are magnified, resulting in slower mobilization, reduced independence, and longer rehabilitation periods. This translates into measurable adverse clinical outcomes, as MSS predicts prolonged hospital length of stay (OR: 2.73), higher 6-month rehospitalization rate (OR: 7.64), and increased 6-month mortality (OR: 1.15), with effect sizes consistently exceeding either condition alone (Sousa et al., 2022[109]). Among older patients undergoing radical gastrectomy, MSS was associated with the lowest HGS, third lumbar vertebra skeletal muscle index, highest rates of total, surgical, and medical complications, and the highest postoperative hospital length of stay, and hospitalization costs compared to single conditions (Chen et al., 2022[23]).

Further, prospective evidence across clinical and community settings has consistently demonstrated that MSS confers a substantially higher mortality risk than either condition alone. In hospitalized older adults (n = 350; mean age: 77.2±7.6 years; follow-up: 2 years), MSS was associated with a hazard ratio (HR) of 19.8 for all-cause mortality, compared with HRs of 3.2 and 2.7 for sarcopenia and malnutrition, respectively (Gümüşsoy et al., 2021[52]). Similarly, in acute geriatric inpatients (n = 453; mean age: 78.3±5.9 years; follow-up 3 years), MSS or the combination of malnutrition risk and sarcopenia were associated with more than a fourfold increase in mortality (HR: 4.78; 95 % CI, 2.09-10.97; and HR: 4.25; 95 % CI, 2.22-8.12), whereas malnutrition risk without sarcopenia doubled mortality risk (HR: 2.41; 95 % CI, 1.32-4.39) (Hu et al., 2017[58]). Population-based data further corroborate these findings. In the National Health and Nutrition Examination Survey cohort (n = 12,469; mean age: 67.5±8.3 years; follow-up of 9-16 years), MSS conferred the highest mortality risk, with total mortality HR 2.66 (95 % CI, 1.89-3.74) and cardiovascular mortality HR 3.56 (95 % CI, 1.17-10.84), whereas isolated sarcopenia or malnutrition had smaller effects (HR 1.62; 95 % CI, 1.28-2.06 and HR 1.28; 95 % CI, 1.03-1.58, respectively) (Chen et al., 2022[22]). Meta-analytic synthesis has further confirmed these findings, showing that MSS consistently predicts greater all-cause mortality among older adults (mean age: > 50years; pooled HR: 4.04; 95 % CI, 1.36-11.94) compared to sarcopenia alone (Prokopidis et al., 2025[96]).

Collectively, these data highlight that MSS represents a synergistic risk factor, wherein concurrent impairments in nutritional and musculoskeletal status amplify long-term mortality risk across diverse populations and follow-up intervals ranging from two to sixteen years.

## 4. Countermeasures

### 4.1 Early screening and diagnosis

Early identification of MSS is essential, as it enables timely and targeted interventions aimed at slowing disease progression, preserving functional independence, and reducing mortality. Current screening and diagnostic approaches, however, focus primarily on malnutrition or sarcopenia individually, and no standardized framework specifically exists for MSS.

For sarcopenia, the European Working Group on Sarcopenia in Older People 2 (EWGSOP2) recommends initial risk screening using the SARC-F (Strength, Assistance with walking, Rise from a chair, Climb stairs, Falls) questionnaire, a brief self-reported instrument assessing functional limitations related to muscle strength and mobility (Cruz-Jentoft et al., 2019[31]). Its simplicity allows rapid implementation in clinical practice. However, while specificity is high, sensitivity is low-to-moderate, meaning that the tool primarily identifies individuals with more advanced stages of sarcopenia. Patients identified as at risk should subsequently undergo objective assessment of muscle strength, commonly using handgrip dynamometry or chair-stand tests, which are strongly predictive of prolonged hospital stays, increased functional limitations, poor quality of life, and death (Cruz-Jentoft et al., 2019[31]). According to the EWGSOP2, low muscle strength defines probable sarcopenia, with a definitive diagnosis requiring evidence of reduced muscle quantity or quality, while impaired physical performance determines disease severity and guides clinical management.

Parallel to sarcopenia screening, systematic identification of malnutrition is recommended. Several tools have been developed for use in older adults, among which the Mini Nutritional Assessment (MNA) is one of the most widely used instruments (Corcoran et al., 2019[27]). While the MNA offers broad coverage, it may overestimate malnutrition risk, making screening alone unreliable for diagnosis and necessitating a structured assessment (Cereda, 2012[20]). Therefore, complementing MNA screening with GLIM criteria, which provide a standardized, evidence-based framework for diagnosis of malnutrition (de van der Schueren et al., 2020[33]) is necessary.

The GLIM framework proposes a two-step model consisting of initial risk screening using any validated tool, followed by a diagnostic assessment and severity grading (Cederholm et al., 2019[18], 2025[19]). Diagnosis requires the presence of at least one phenotypic criterion, including unintentional weight loss, low body mass index, or reduced muscle mass, together with one etiologic criterion such as reduced food intake/assimilation or the presence of disease burden/inflammation. Among these indicators, reduced muscle mass is strongly supported as a central phenotypic criterion, reflecting the close pathophysiological relationship between malnutrition and skeletal muscle depletion. Although assessment of muscle strength is not part of the formal GLIM diagnostic algorithm, it is recommended as a supportive measure, particularly when sarcopenia is suspected (Cederholm et al., 2019[18], 2025[19]).

Screening for and managing malnutrition is recognized as a crucial early step in the identification and treatment of sarcopenia (Vandewoude et al., 2012[118]), as early nutritional deficits can directly contribute to muscle loss. This link is supported by longitudinal cohort studies demonstrating the prognostic value of GLIM-defined malnutrition for the development of sarcopenia, and consequently for MSS. In community-dwelling older adults, malnutrition according to the GLIM criteria strongly predicted incident sarcopenia over a 14-year follow-up period (Yeung et al., 2021[133]). Similarly, in the SarcoPhAge cohort, individuals classified as malnourished exhibited an approximately threefold higher risk of developing sarcopenia or severe sarcopenia over four years compared with participants with adequate nutritional status (Beaudart et al., 2019[6]). Importantly, subsequent analyses demonstrated that while the GLIM criteria reliably predicted incident sarcopenia, traditional screening tools such as the long and short form of the MNA did not (Lengelé et al., 2021[71]).

Taken together, these findings indicate that screening for malnutrition alone is insufficient to identify older adults at high risk of sarcopenia. Early, integrated assessment strategies that combine nutritional evaluation with objective measures of muscle mass and strength, supported by ongoing monitoring, are essential to track disease progression and optimize management of MSS.

### 4.2 Nutrition and exercise

The concurrence of malnutrition and sarcopenia defines a particularly vulnerable subgroup of older adults, often characterized by multimorbidity, frailty, and pronounced functional decline. Effective management therefore requires highly individualized and interdisciplinary approaches that are carefully aligned with the patient's physiological capacity, safety profile, and treatment tolerance. While direct evidence for interventions specifically targeting MSS is lacking, current nutritional and exercise recommendations established for malnutrition alone and sarcopenia alone may offer a pragmatic provisional framework for clinical practice. Nonetheless, their application should remain nuanced and guided by clinical judgment, recognizing that the pathophysiological interplay between malnutrition and sarcopenia may modify therapeutic responsiveness and ultimately demand condition-specific adaptations as empirical data emerge.

#### Nutritional therapy

The European Society for Clinical Nutrition and Metabolism guidelines recommend an average energy intake of approximately 30 kcal/kg body weight/day for older adults to prevent negative energy balance and muscle catabolism, with adjustments according to nutritional status, physical activity, disease burden, and tolerance (Volkert et al., 2022[123]). Higher intakes of 32-38 kcal/kg/day are suggested for individuals with underweight to support weight maintenance or regain (Volkert et al., 2022[123]). Protein intake of 1.0-1.2 g/kg/day is advised for healthy older adults (≥ 65 years), with escalation to 1.2-1.5 g/kg/day during acute catabolic states, such as illness, inflammation, or prolonged bed rest, while taking renal function and individual tolerance into account (Bauer et al., 2013[3]; Deutz et al., 2014[38]; Volkert et al., 2022[123]). Whether these targets are sufficient for individuals with MSS remains unclear, as the coexistence of malnutrition and sarcopenia may amplify anabolic resistance and metabolic demands, potentially necessitating higher or individualized protein and energy intake.

Protein intake should emphasize high-biological-value sources, particularly whey protein, which provides a rich profile of essential amino acids (EAAs) and is rapidly digested and absorbed, eliciting superior anabolic stimulation compared with soy, casein, or hydrolyzed casein (Bauer et al., 2013[3]). Among EAAs, leucine is considered as the most potent amino acid for promoting muscle growth, as it modulates protein turnover in skeletal muscle by both stimulating MPS and suppressing MPD (Nie et al., 2018[87]; Harris et al., 2025[53]). To counteract anabolic resistance and optimize MPS, 25-30 g of high-quality protein per meal, providing 2.8-3 g of leucine, at least twice daily, is recommended (Bauer et al., 2013[3]; Harris et al., 2025[53]). This approach yields minimum daily leucine intakes of approximately 78.5 mg/kg body weight, substantially exceeding the current recommended allowance of 34 mg/kg body weight per day (Harris et al., 2025[53]).

Oral nutritional supplementation (ONS) is indicated when dietary intake is insufficient. High-protein ONS (> 20 % of total energy from protein) have been shown to increase total energy and protein intake, supporting gains in body weight and muscle strength, while reducing complications, hospital length of stay, and readmissions (Cawood et al., 2012[16]; Elia et al., 2016[43][44]). In older adults with both malnutrition and sarcopenia, daily high-quality ONS improved leg strength, muscle quality, HGS, and gait speed over 24 weeks, with larger gains in mild-to-moderate sarcopenia and delayed responses in severe cases (Cramer et al., 2016[30]). Similar benefits were observed in patients with gastric cancer and MSS, where ONS preserved body weight and suggested trends toward improved functional outcomes (Wu et al., 2025[130]). These findings highlight the potential of targeted nutritional support in enhancing physical performance in MSS, particularly when sarcopenia is less advanced.

Leucine supplementation has been shown to enhance MPS via the mTOR pathway (Wilkinson et al., 2013[129]), even when protein intake is low. Evidence from older adults (≥ 60 years) with sarcopenia, however, indicates that leucine alone without exercise, does not significantly improve muscle mass, strength, or function (Conde Maldonado et al., 2022[26]). Nevertheless, when combined with exercise, particularly resistance training (RT) or delivered together with whey protein and vitamin D, it significantly increases muscle mass, strength, and functional capacity, producing clinically meaningful outcomes including reduced comorbidity burden, fewer falls, and lower fracture risk (Conde Maldonado et al., 2022[26]).

For β-hydroxy-β-methylbutyrate (β-HMB), an active metabolite of leucine, evidence from an umbrella review of 15 systematic reviews indicates only a minor and inconsistent support, whether administered as part of an ONS, as a stand-alone supplement, or combined with other amino acids, for preserving lean soft tissue mass or improving muscle strength (Phillips et al., 2022[92]). Furthermore, no clear additive benefit has been observed when β-HMB is combined with exercise interventions (Gielen et al., 2021[51]; Phillips et al., 2022[92]).

While nutritional deficiencies should always be corrected, monotherapy with individual nutrients is not supported by evidence as a means to improve muscle function, although some single nutrients deserve special attention given their specific roles in muscle metabolism. Vitamin D deficiency e.g. is highly prevalent in older adults and has been associated with reduced muscle strength, lower muscle mass, and poorer physical performance across observational studies. However, evidence from intervention studies remains inconsistent. While some systematic reviews and meta-analyses report small but significant improvements in muscle strength (Beaudart et al., 2014[5]), others have found no measurable benefits (Rosendahl-Riise et al., 2017[98]; Abshirini et al., 2020[1]; Prokopidis et al., 2022[94]). Similarly, findings regarding appendicular lean soft tissue and physical performance vary considerably, with studies reporting positive (Rosendahl-Riise et al., 2017[98]; Harrison and Ghosh, 2023[54]), negative (Abshirini et al., 2020[1]; Prokopidis et al., 2022[94]), or neutral effects (Widajanti et al., 2024[127]). These discrepancies likely reflect differences in study populations, baseline vitamin D status, and supplementation dosing. Some evidence suggests that individuals with low baseline vitamin D concentrations may derive greater benefit from supplementation (Stockton et al., 2011[111]; Beaudart et al., 2014[5]; Abshirini et al., 2020[1]), although this finding has not been consistently replicated (Rosendahl-Riise et al., 2017[98]). Current clinical practice guidelines therefore position vitamin D as an adjunct to high-protein nutritional strategies and RT in sarcopenia management (Dent et al., 2018[36]).

#### Exercise

While exercise interventions have emerged as the most effective non-pharmacological approach for managing sarcopenia, with RT identified as first-line treatment (Deutz et al., 2014[38]; Dent et al., 2018[36]), its implementation in patients with MSS may be constrained by pronounced clinical vulnerability. During the initial phase, preservation of mobility and the prevention of further functional decline is mandated.

Early mobilization and individually tailored physiotherapy progressing from range-of-motion exercises to postural control and gait training may help maintain neuromuscular function and limit disuse-related muscle loss. In parallel, adequate pain management and optimized treatment of comorbidities, with careful medication review, could further support the patient's capacity to participate in rehabilitation. Alternative modalities, such as neuromuscular electrical stimulation, may be considered to prevent muscle atrophy when conventional mobilization proves impossible, although supporting evidence remains limited and heterogeneous (Nonoyama et al., 2022[88]; Deshmukh et al., 2025[37]).

Depending on an individual's functional capacity and tolerance, balance and aerobic exercises may serve as complementary interventions. Although their isolated effects have shown only negligible effects on muscle strength and physical performance compared with RT in older adults with sarcopenia, both modalities confer important benefits. Balance training enhances postural stability, coordination, proprioception, and reflex responses, thereby reducing fall risk while engaging multiple muscle groups, including stabilizers, to promote muscular endurance (Pedersen, 2019[91]; Moretti et al., 2025[83]). Aerobic exercise (e.g., walking, cycling, swimming) enhances cardiovascular capacity and muscular endurance, supports metabolic health through improved insulin sensitivity, and may facilitate long-term adherence to physical activity programs (Ziaaldini et al., 2017[138]).

Progressive RT should be initiated only after a comprehensive assessment of health status and physical performance to exclude contraindications and determine exercise type, intensity, and starting level. As described above, in patients with MSS, particular attention should be paid to nutritional status prior to initiating higher-intensity exercise, as inadequate energy and protein intake may limit adaptive responses to training. Nevertheless, even among highly vulnerable older adults, skeletal muscle retains remarkable plasticity. The landmark study by Fiatarone and colleagues (1994[46]) demonstrated that 10 weeks of progressive high-intensity RT produced substantial gains in muscle strength and physical performance among nursing home residents with frailty aged up to 98 years (mean age: 87 years). Although sarcopenia and malnutrition were not formally assessed, the advanced age, institutionalization, and frailty among participants suggest that a subset likely exhibited MSS.

Combining RT with complementary exercise modalities appears beneficial, as shown in a network meta-analysis of 42 randomized controlled trials including 3,728 older adults with sarcopenia (mean age: 72.9 years). RT combined with aerobic and/or balance training ranked highest for improving physical function, outperforming isolated RT or other single-modality interventions (Shen et al., 2023[105]). Multimodal exercise programs, with or without nutritional support, were also associated with most effective improvements in quality of life. Consistent with these findings, the SPRINTT trial (n = 1,519; mean age: 78.9 years) demonstrated that a multicomponent intervention combining structured physical activity with individualized nutritional counselling significantly reduced the incidence of mobility disability in older adults with physical frailty and sarcopenia compared with lifestyle education alone (Bernabei et al., 2022[8]).

In summary, evidence-based intervention strategies specifically targeting MSS are currently lacking. The approaches outlined in this chapter therefore represent conceptual frameworks extrapolated from research on isolated malnutrition and sarcopenia, rather than MSS-specific therapeutic recommendations. Given the likely synergistic interaction between the two conditions, their coexistence may impose physiological demands distinct from those observed in either disorder alone, suggesting the future need for tailored treatment adaptations once direct clinical evidence emerges.

## 5. Conclusion

Malnutrition and sarcopenia frequently co-occur in older adults and interact in a vicious cycle, in which each condition can induce or exacerbate the other, a phenomenon proposed as MSS. Whether MSS constitutes a unique syndrome or merely reflects the overlap of two closely related conditions remains debated. Regardless, the interplay of these conditions appears to synergistically worsen adverse outcomes, particularly mortality, and contributes to substantial cumulative healthcare costs. Recognizing this clinical pattern serves the pragmatic purpose of identifying vulnerable older adults who may benefit from comprehensive and simultaneous nutritional and functional interventions.

While validated screening tools and management guidelines exist for malnutrition and sarcopenia individually, no standardized MSS-specific framework currently addresses their intersection, highlighting a critical gap in geriatric care. Further research is therefore warranted to develop simple, validated MSS-specific screening strategies, define unified diagnostic criteria, and establish evidence-based clinical management guidelines, tailored to the patient's functional capacity and safety profile, aimed at disrupting the vicious cycle between malnutrition and sarcopenia and improving patient outcomes.

## Declaration

### Conflict of interest

The authors declare that they have no conflict of interest.

### Funding

This research received no external funding.

### Artificial Intelligence (AI) - assisted technology

AI-assisted tools were used only to improve grammar and readability of the human-generated text. No AI tools were used for conceptualization, scientific content generation, data interpretation, conclusion drawing, and preparation of the graphical abstract and figures. The authors have thoroughly proofread the manuscript and take full responsibility for the accuracy, integrity, and originality of its content.

### Author contributions

**Figure 1:**
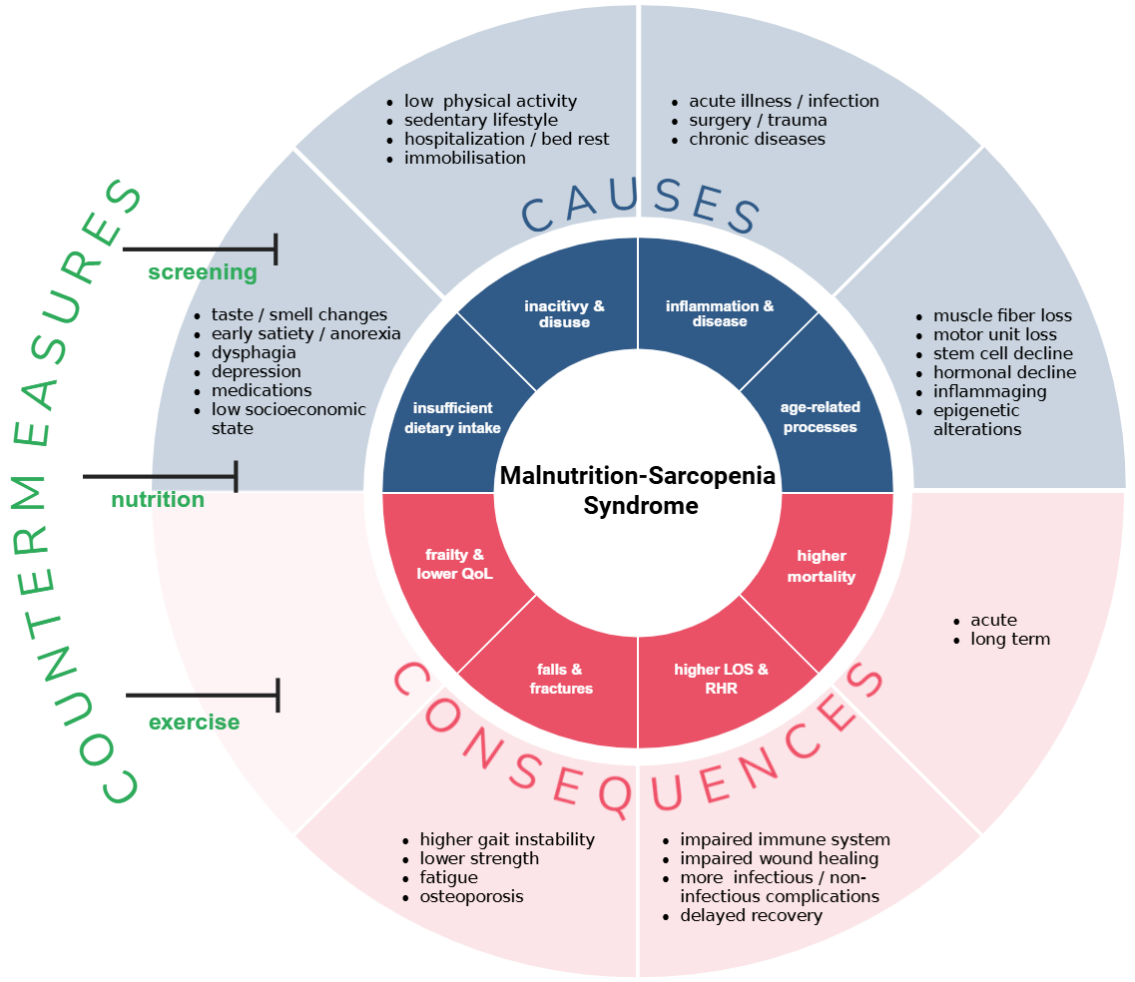
Graphical Abstract. Causes, consequences, and countermeasures of the Malnutrition-Sarcopenia Syndrome in older adults. All factors, whether located in the outer or inner ring, are considered potential determinants (light and dark blue) or consequences (light and dark red) of the MSS, respectively. Abbreviations: LOS, hospital length of stay; QoL, quality of life; RHR, rehospitalization rate. Created in BioRender. Walowski, C. (2026) https://BioRender.com/jwkoj1g

**Figure 2:**
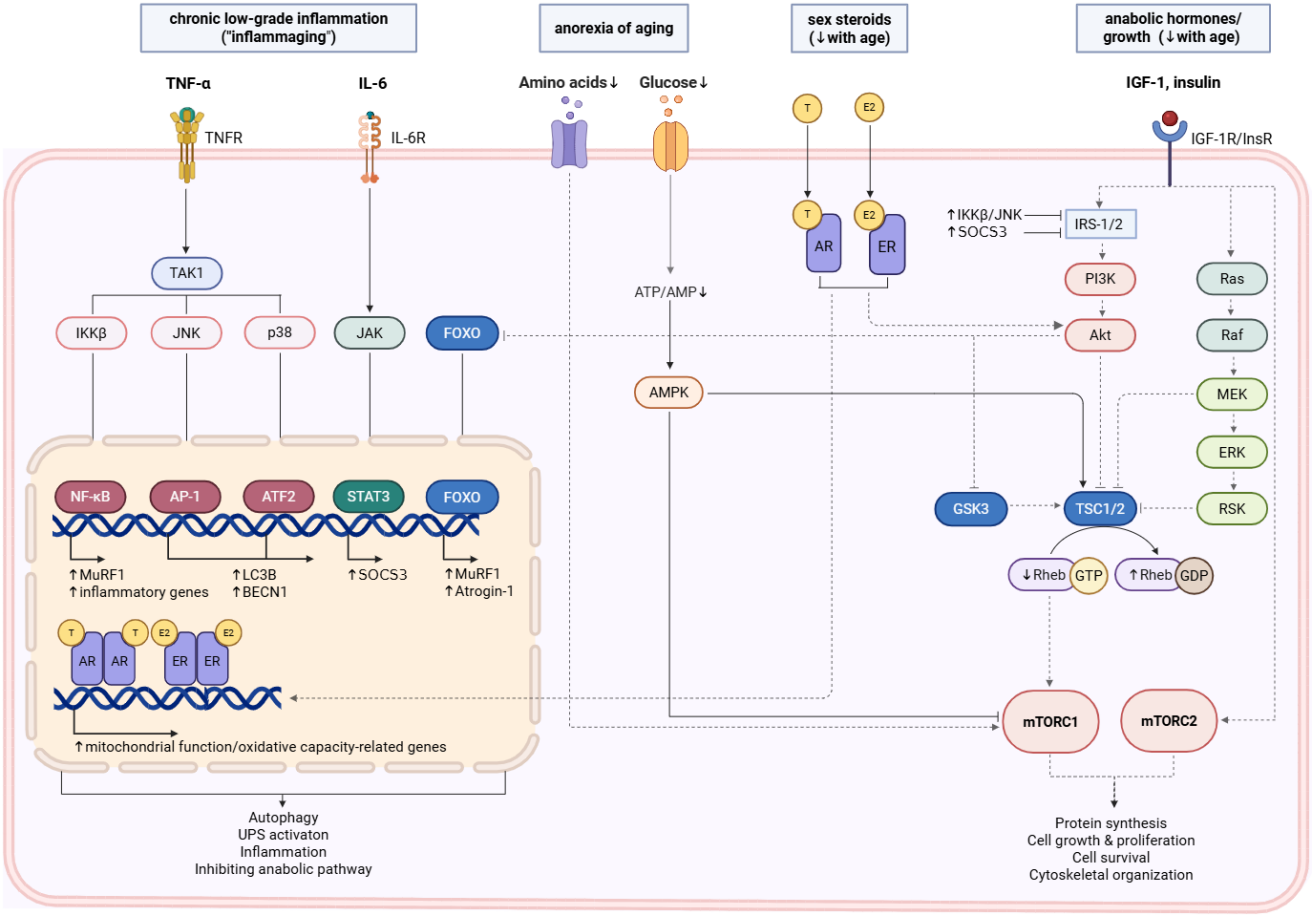
Conceptual model of the potential effects of age-related intrinsic factors on skeletal muscle signaling pathways. Solid lines ending in an arrowhead: activation; dashed lines ending in an arrowhead: attenuated activation; solid lines ending in a blunt bar (-|): inhibition; dashed lines ending in a blunt bar: attenuated inhibition; up and down arrows: relative increase or decrease with aging. Abbreviations: Akt, protein kinase B; AMP, adenosine monophosphate; AMPK, AMP-activated protein kinase; AP-1, activator protein 1; AR, androgen receptor; ATF2, activating transcription factor 2; ATP, adenosine triphosphate; BECN1, Beclin 1; E2, estradiol; ER, estrogen receptor; ERK, extracellular signal-regulated kinase; FOXO, forkhead box O proteins; GDP, guanosine diphosphate; GSK3, glycogen synthase kinase-3; GTP, guanosine triphosphate; IGF-1, insulin-like growth factor 1; IGF-1R, insulin-like growth factor 1 receptor; IKKβ, IκB kinase beta; IL-6, interleukin 6; IL-6R, interleukin 6 receptor; InsR, insulin receptor; IRS-1/2, insulin receptor substrates 1/2; JAK, Janus kinase; JNK, c-Jun N-terminal kinase; LC3B, microtubule-associated protein 1 light chain 3 beta; MEK, mitogen-activated protein kinase kinase; mTORC1, mammalian target of rapamycin complex 1; mTORC2, mammalian target of rapamycin complex 2; MuRF-1, muscle RING-finger protein 1; NF-κB, nuclear factor kappa-light-chain-enhancer of activated B cells; PI3K, phosphatidylinositol 3-kinase; Raf, rapidly accelerated fibrosarcoma; Ras, rat sarcoma; Rheb, Ras homolog enriched in brain; RSK, ribosomal S6 kinase; SOCS3, suppressor of cytokine signaling 3; STAT3, signal transducer and activator of transcription 3; T, testosterone; TAK1, transforming growth factor β-activated kinase 1; TNF-α, tumor necrosis factor alpha; TNFR, tumor necrosis factor receptor; TSC1/2, tuberous sclerosis complex 1/2; UPS, ubiquitin-proteasome system. Created in BioRender. Walowski, C. (2026) https://BioRender.com/eyavzw0

**Figure 3:**
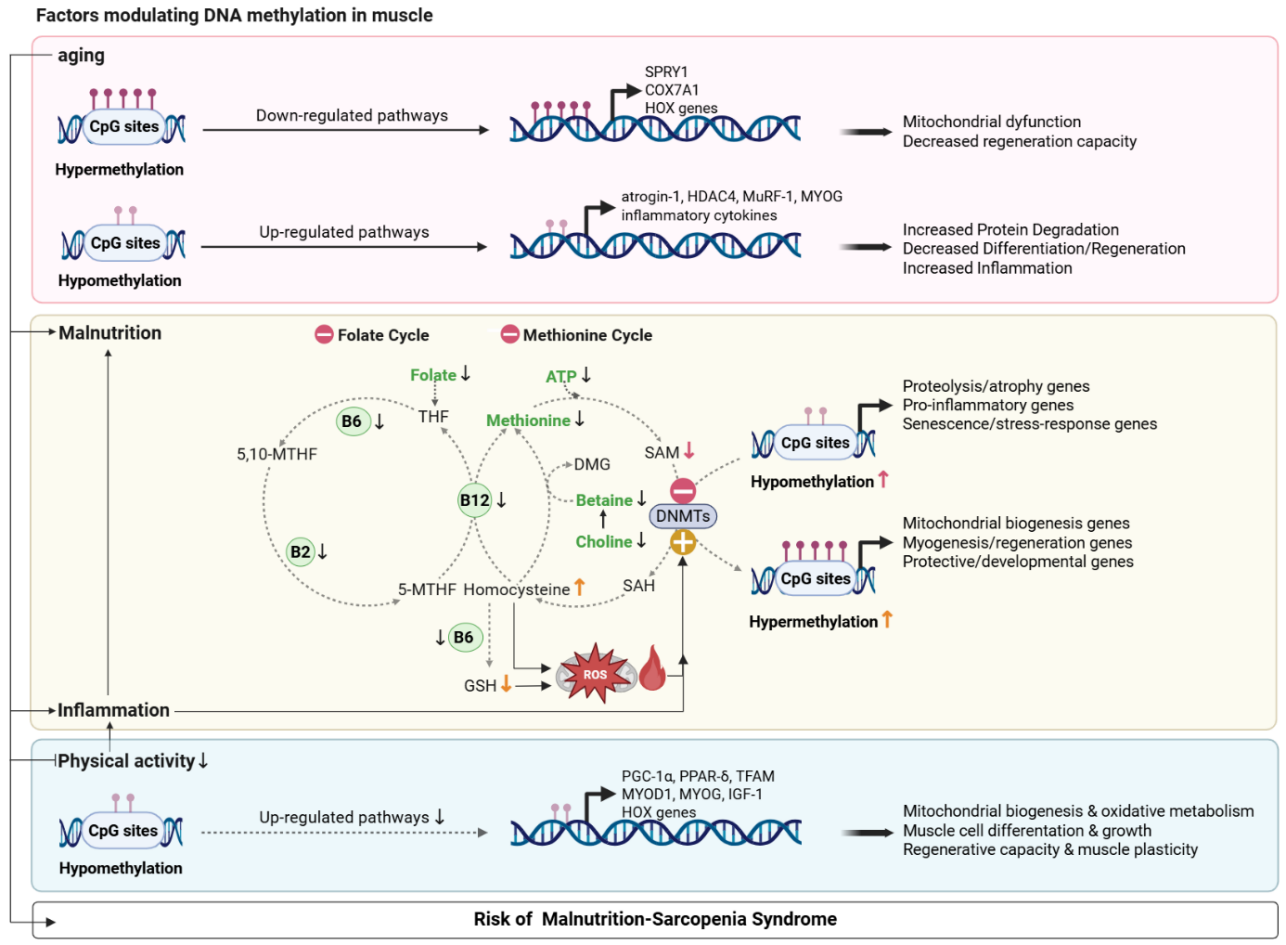
Conceptual model illustrating potential factors and mechanisms by which DNA methylation drift may contribute to sarcopenia and, subsequently, to the Malnutrition-Sarcopenia Syndrome during aging. Solid arrows: lead to/promote; dashed arrows: attenuated pathway; up and down arrows: relative increase or decrease; red arrows: SAM-deficient (hypomethylation) pathway; yellow arrows: homocysteine and GSH-ROS-inflammation (hypermethylation) pathway. Abbreviations: ATP, adenosine triphosphate; B2, riboflavin; B6, pyridoxine; B12, cobalamin; COX7A1, cytochrome c oxidase subunit 7A1; CpG, cytosine-phosphate-guanine dinucleotide; DMG, dimethylglycine; DNMTs, DNA methyltransferases; GSH, glutathione; HDAC4, histone deacetylase 4; HOX, homeobox; IGF-1, insulin-like growth factor 1; MTHF, methyltetrahydrofolate; MuRF-1, muscle RING-finger 1; MYOD1, myogenic differentiation 1; MYOG, myogenin; PGC-1α, peroxisome proliferator-activated receptor gamma coactivator 1-alpha; PPAR-δ, peroxisome proliferator-activated receptor delta; ROS, reactive oxygen species; SAH, S-adenosyl-L-homocysteine; SAM, S-adenosyl-L-methionine; SPRY1, Sprouty RTK signaling antagonist 1; TFAM, mitochondrial transcription factor A; THF, tetrahydrofolate. Created in BioRender. Walowski, C. (2026) https://BioRender.com/z8h7got

C.O.W. contributed to writing - original draft, visualization (figures 1 to 3(Fig. 1)(Fig. 2)(Fig. 3)), and writing - review & editing. K.N. contributed to conceptualization, visualization (figure 1(Fig. 1)), and writing - review & editing. All authors have read and agreed to the published version of the manuscript.
